# Averting Behavior Framework for Perceived Risk of *Yersinia enterocolitica* Infections

**DOI:** 10.1155/2012/725373

**Published:** 2012-03-07

**Authors:** Sonia N. Aziz, Khwaja M. S. Aziz

**Affiliations:** ^1^Moravian College, 210 Comenius Hall, Bethlehem, PA 18018, USA; ^2^Bangladesh Academy of Sciences, Dhaka 1207, Bangladesh

## Abstract

The focus of this research is to present a theoretical model of averting actions that households take to avoid exposure to *Yersinia enterocolitica* in contaminated food. The cost of illness approach only takes into account the value of a cure, while the averting behavior approach can estimate the value of preventing the illness. The household, rather than the individual, is the unit of analysis in this model, where one household member is primarily responsible for procuring uncontaminated food for their family. Since children are particularly susceptible and live with parents who are primary decision makers for sustenance, the designated household head makes the choices that are investigated in this paper. This model uses constrained optimization to characterize activities that may offer protection from exposure to *Yersinia enterocolitica* contaminated food. A representative household decision maker is assumed to allocate family resources to maximize utility of an altruistic parent, an assumption used in most research involving economics of the family.

Yersiniosis remains a public health hazard due to exposure to contaminated food and human to human or zoonotic infections. *Yersinia enterocolitica* is an important cause of yersiniosis in humans and animals; its epidemiology remains yet to be fully understood and exposure to it is a growing food safety concern [1–5]. There are a number of recent reviews published on specific aspects of *Y. enterocolitica*, and while some of these studies investigate incidence rates, true incidence in developed and developing countries remain unknown [1, 6–10]. One of the most frequent outcomes of *Y. enterocolitica *is possibly diarrhea as exemplified by a study in Poland [11]. A study on methods of monitoring trends in incidence of foodborne diseases in the United States is a welcome instrument in the estimation of incidence of *Y. enterocolitica* and other pathogens [12]. Studies of incidence, combined with studies investigating behaviors of individuals responding to information of incidence and risk levels of *Y. enterocolitica* can be useful for public health mitigation policies. In this paper we discuss a behavioral model with a focus on avoiding health hazards associated with exposure to *Y. enterocolitica. *The paper is theoretical and the conceptual model presented here is not showcased with data. The theoretical framework easily lends itself to application subject to availability of secondary data. One major thrust of the theoretical discussion revolves around the heuristic notion “an ounce of prevention is worth a pound of cure.”

As infection from *Y. enterocolitica * is typically contracted through eating contaminated food, an examination of averting behavior may help identify the burden of disease better than the cost of illness approach. Any action taken by an individual to avoid an illness is considered averting behavior. Cost of illness is typically and widely used in public health policy analysis and includes both direct and indirect costs. Whether analyses incorporate direct or indirect costs, cost of illness studies focus on estimating the costs of cure. Direct costs take into account the cost of resources used to treat illness while indirect cost measures the value of resources foregone due to the illness. Cost of illness studies are generally considered underestimates as they do not take into account two things. Cost of illness studies do not take into account pain and suffering as one endures an illness. Also, the notion that cost of illness studies provide a lower bound for the value of avoiding the illness is supported by the heuristic notion that a person is willing to pay significantly more to avoid an illness (values toward prevention) than to become ill in the first place (values toward cure). 

Averting behavior models take into account any action or expenditures that individuals undertake to avoid an illness. Averting behavior models are protective expenditures or actions that individuals undertake to avoid exposure to any undesirable outcome (e.g., pollution, illness, death). This is an approach that says that the value of a small reduction in health state can in principle be measured by the amount an individual is willing to spend on some defensive or averting action to prevent it. The assumption in the averting behavior approach in this study is that people make choices in order to maximize their level of well-being when faced with increased health risks associated with exposure to contaminated food [13]. The notion here is that individuals present subjective individual preferences toward avoiding the illness. Their subjective individual valuations are measured and taken as given. The main hypothesis here is that individuals value their health and make optimizing choices to maximize their well-being subject to certain constraints. Assuming only monetary choices (averting behavior studies do not preclude individual actions, but in this note we are assuming expenditures only), individuals make these choices subject to their budget constraints. Also, individuals make these choices about risk mitigation without knowing whether they or a member of their household will be ill: choices are predicated on perceived risk of *Y*. *enterocolitica*. 

Along these lines, we present a simple model of choice under uncertainty. Most behavioral economics use the notion of utility, where utility represents an individuals' level of well-being. In economic theory, individuals are assumed to take actions in order to maximize their level of well-being, and these actions are limited by their resource constraints. Here, we define Utility (*U*_*i*_)  to represent individual well-being. Utility (*U*_*i*_) is assumed to be a function of wealth (*W*), health (*H*), perceived risk levels (*r*), and averting activities (∝). The resource constraint that the individual faces is represented by *W*, which the reader can intuitively take to mean real wealth. *W* is what remains of individual wealth after costs of actions undertaken to avoid the risk are taken into consideration. If the level of action is represented by ∝, and if it costs the individual a price *p* per unit of the averting activity, we can represent the remaining amount of real wealth by removing the total cost of the averting activity *p*∝ from initial wealth levels (*W*_0_). Since individuals do not know whether they will be ill from contaminated food, their utility (of maintaining health and well-being) is not known with certainty. In order to deal with this, utility must be cast in a framework consistent with the probability of becoming ill [14]. Therefore, utility is weighted by probability of health state where the simplest possible health state is examined. Health states are indexed by *i*, where *i* goes from 1 to 2. Health states can theoretically be indexed by *i* going from 1 through *n* possible future states of health. For ease of exposition, *i* is suppressed to only two states indicating the probability that one can either be healthy (*i* = 1) or ill (*i* = 2) from *Y*. *enterocolitica* contaminated food. Also please note that wealth, health, and risk levels are themselves functions of the level of averting activity. The arguments for these variables are suppressed in the objective function for notational simplicity. Maximizing utility subject to the budget constraint is then expressed as follows:

(1) $$\begin{array}{cc} \underset{\propto }{\mathrm{Max}} & \;\overset{2}{\underset{i=1}{\sum }}\pi_{i}U_{i}\left(W,H,r,\propto \right),\\ \text{Subject}\;\text{to} & \;W=W_{0}-p\propto , \end{array}$$

where *π*_*i*_ is probability of being in health state *i*, *U*_*i*_ is utility (well-being) in health state *i*, *W* is wealth, *H* is individual health, *r* is perceived ambient risk from *Y. enterocolitica, *∝ is averting activity, *W*_0_ is initial wealth level, and *p* is price of averting activity.

Assuming the simplest possible form of utility function (one in which additional utility from health, wealth, and from reducing risk are additive), we can take first-order conditions. Translated in discrete terms, we are finding the point at which an individual can choose the maximum amount of utility allowed by their budget constraint. In continuous terms, when we take the first-order conditions with respect to the averting activity ∝ and perceived risk reductions *r*, we find a simple efficiency condition:

(2) $$\overset{2}{\underset{i=1}{\sum }}\pi \left(\frac{\partial U_{i}}{\partial \alpha }+\frac{\partial W}{\partial \alpha }+\frac{\partial r}{\partial \alpha }\right)=p.$$

The condition above simply states that averting activity will continue until the incremental benefits perceived from averting (left hand side) equal the incremental cost of averting (right hand side). This condition along with an individual's action to reduce risk levels (represented mathematically by first order conditions optimizing over reducing risk levels *r*) form the estimated value for averting *Y. enterocolitica. *The first order conditions over reducing risk level *r* are of the form:

(3) $$\overset{2}{\underset{i=1}{\sum }}\pi \left(\frac{\partial U_{i}}{\partial r}+\frac{\partial W}{\partial r}+\frac{\partial r}{\partial r}\right)=0.$$

Please see the appendix for derivations of the above equations. This first-order condition represents the individuals choice to reduce risk from contracting infection from *Y. enterocolitica. *Both conditions above intuitively represent individuals choice, and most importantly, value from averting infection from *Y. enterocolitica*. This paper does not apply the theoretical model above to data, primarily because no secondary data is available, and therefore we cannot comment on the value of using averting behavior versus cost of illness for *Y. enterocolitica. *While the empirical studies comparing WTP (willingness to pay) estimates with cost of illness (COI) estimates are few and far between, the comparisons that have been done show that WTP is at least 1.6 to 8.0 times larger than COI., [15–19]. One recent study [20] computed and compared willingness to pay for avoiding *shigellosis* to the cost of illness of *shigellosis. *The evidence on whether WTP estimates are higher than COI estimates were mixed in the paper. Key messages from the study include the following:

*“For evaluating the benefits of public programmes to control shigellosis, the use of the conventional and convenient ex post COI figures for adults instead of ex post WTP measures may yield acceptable measures of the welfare impacts of reducing disease risk. However, the use of ex post COI as the estimate of the welfare impact of risk-reducing policies is likely to underestimate the benefits of programmes to prevent shigellosis in children.”*

While cost of illness approaches to prevent *Y. enterocolitica *in adults may suffice, public health mitigation policy makers may wish to focus on using methodologies such as averting behavior to estimate values for avoided illnesses in children. A direction for future research using the averting behavior model developed in this paper involves collecting primary data in order to test the theoretical model. A swift review of the studies cited, and future work with an applied averting behavior model may support the following notion “an ounce of prevention is worth a pound of cure, in the case of children.” Perhaps this reflects the notion that in the case of adults, taking a calculated risk may be more palatable than in the case of children. 

## Appendix

Considering (1) we have the following.

Assuming an additively separable utility function the following first-order conditions with respect to the averting activity ∝ follow.

Let

(A.1) $$\begin{array}{cc} EU & =\overset{2}{\underset{i=1}{\sum }}\pi \left(\frac{\partial U_{i}}{\partial \alpha }+\frac{\partial W}{\partial \alpha }+\frac{\partial r}{\partial \alpha }\right) \end{array}.$$

Taking first-order conditions from the utility function with respect to ∝ yeilds

(A.2) $$\begin{array}{cc} \frac{\partial EU}{\partial \propto } & =\overset{2}{\underset{i=1}{\sum }}\pi \left(\frac{\partial U_{i}}{\partial \alpha }+\frac{\partial W}{\partial \alpha }+\frac{\partial r}{\partial \alpha }\right). \end{array}$$

Taking first-order conditions from the budget constraint with respect to ∝ yeilds

(A.3) $$\frac{\partial W}{\partial \propto }=p.$$

Equations (A.2) and (A.3) taken together represent the point of utility maximization where the slope of the budget constraint ∂*W*/∂∝ must be equal to the tangent to the utility function ∂*EU*/∂∝.

Therefore,

(A.4) $$\overset{2}{\underset{i=1}{\sum }}\pi \left(\frac{\partial U_{i}}{\partial \alpha }+\frac{\partial W}{\partial \alpha }+\frac{\partial r}{\partial \alpha }\right)=p.$$

Equation (A.4) simply states that averting activity will continue until the marginal benefits perceived from averting (left-hand side) equal the marginal cost of averting (right-hand side). This condition along with first-order conditions optimizing over reducing risk levels *r* form the estimated value for averting *Y*. *enterocolitica*.
